# Spatial *k*-dispersion engineering of spoof surface plasmon polaritons for customized absorption

**DOI:** 10.1038/srep29429

**Published:** 2016-07-08

**Authors:** Yongqiang Pang, Jiafu Wang, Hua Ma, Mingde Feng, Yongfeng Li, Zhuo Xu, Song Xia, Shaobo Qu

**Affiliations:** 1 School of Electronics & Information Engineering, Xi’an Jiaotong University, Xi’an, Shanxi 710049, People’s Republic of China; 2 College of Science, Air Force Engineering University, Xi’an, Shaanxi 710051, People’s Republic of China

## Abstract

Absorption of electromagnetic waves in a medium is generally manipulated by controlling the frequency dispersion of constitutive parameters. However, it is still challenging to gain the desired constitutive parameters for customized absorption over a broad frequency range. Here, by virtue of spoof surface plasmonic polaritons (SPPs), we demonstrate capabilities of the spatial *k*-dispersion engineering for producing the customized broadband absorption. Incident waves can be efficiently converted to the spoof SPPs by plasmonic arrays, and their propagation and/or absorption can be controlled by engineering the spatial dispersion of *k*-vector. Based on this feature, we show how such concept is employed to achieve broadband as well as frequency-selective broadband absorptions as examples. It is expected that the proposed concept can be extended to other manipulations of propagating electromagnetic waves over a broad frequency range.

Controlling absorption from a medium illuminated by electromagnetic waves is of great importance in many areas. Particularly, approaches for enhancing absorption have been widely investigated in the last several decades and are believed to be continuing in the future due to the great potential applications in the fields of energy-harvesting devices^1^,^2^,^3^,^4^, thermal emitters/detectors^5^,^6^,^7^, microwave absorbing materials^8^,^9^,^10^,^11^,^12^,^13^, display devices^14^, and so on. The electromagnetic power absorbed in a non-magnetic medium is mainly determined by losses as well as electric field strength, following the relation *P*_abs_ = 1/2(*ωε*″ + *σ*)|*E*|^2^, where *ω* is the angular frequency, *ε*″ is the imaginary part of permittivity, *σ* is the conductivity and *E* is the total electric field^15^. Clearly, increase of the losses (*ε*″ and/or *σ*) and the electric field allows strong absorption, but they are commonly conflicting requirements with the metal in microwave regime as a typical example. As a consequence, various mechanisms for enhancing the total electric field are exploited to achieve strong absorption^2^,^3^,^4^,^5^,^16^,^17^,^18^,^19^,^20^,^21^,^22^,^23^,^24^,^25^,^26^. As an important aspect of this topic, the frequency dispersion of the constitutive parameters is necessary for high-efficiency absorption over a broad frequency band^12^,^27^, but realization of such materials is challenging. Fortunately, recent advantage of metamaterials comprising sub-wavelength inclusions^28^ offers a promising way to manipulate the constitutive parameters with freedom to produce broadband responses. For example, the artificial frequency dispersion engineering of both real and imaginary parts of the effective permittivity of a metamaterial has been demonstrated to achieve broadband absorption^29^.

Here, we introduce a concept on the spatial dispersion engineering of *k*-vector to create customized absorption over a broad frequency band. It is known that the *k*-vector characterizes the spatial variation of the field strength and thus is also called visually as the spatial frequency^30^. The high *k*-vector at a given frequency means that the field is spatially squeezed and thus may be enhanced within a sub-wavelength region. If this condition can be fulfilled, the strong absorption would be achieved by introducing proper losses. Furthermore, absorption can be customized over a broad frequency range by engineering the spatial dispersion of the *k*-vector. From the dispersion relation *k*^2^ = *ω*^2^*εμ* with the permittivity *ε* and the permeability *μ*, we see that the *k*-vector can be engineered with the constitutive parameters of *ε* and *μ*, but the spatial dispersion control of *ε* and *μ* is needed at each frequency^31^. In contrast, it would be an ideal strategy to directly engineer the spatial dispersion of the *k*-vector and this can be easily implemented by virtue of surface plasmon polaritons (SPPs)^32^ of which dispersion relations are controllable. More importantly, the condition of strong absorption can be fulfilled simultaneously due to the sub-wavelength field-confinement effect of SPPs. The SPPs naturally exist at optical frequencies, but suitably structured metamaterials have been developed to excite the spoof SPPs at low frequencies^33^,^34^,^35^. In particular, recent study on the corrugated plasmonic strips with nearly zero thickness^36^,^37^,^38^,^39^,^40^,^41^ is promising for the required structures in this work. We took such derivative structures as an effective validation for the concept of spatial *k*-dispersion engineering for achieving the customized absorption over a broad frequency band, and also implemented experimental demonstrations. It should be emphasized that our goal is not strictly to design a broadband absorber, which have been extensively studied previously^3^,^10^,^12^,^18^,^19^,^20^,^21^,^23^,^24^,^29^. Here we focus on the concept of spatial *k*-dispersion engineering for achieving broadband customable response, and perform the demonstration with absorption as an example.

## Results

### Exploitation of spoof SSPs for absorption

**Figure 1:**
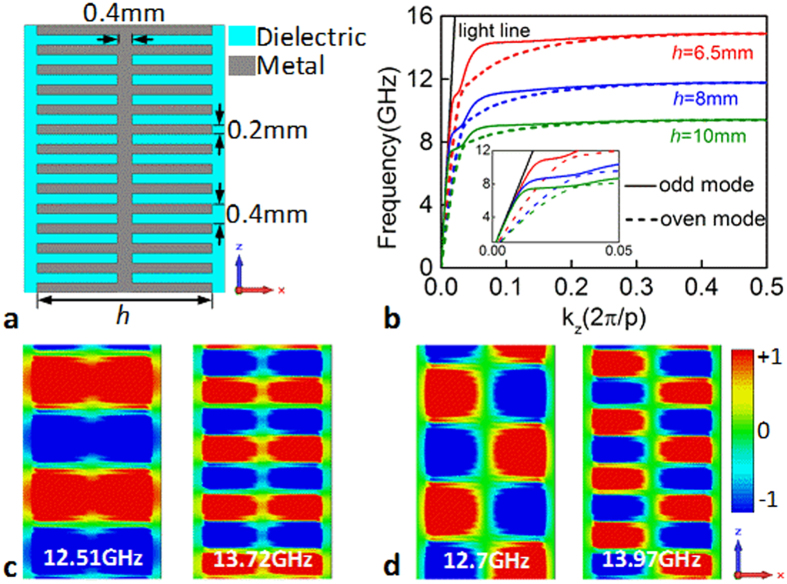
Plasmonic structure made of a corrugated metal strips adhered on an ultrathin dielectric layer for exciting the spoof SPPs. (**a**) Schematic illustration of the plasmonic structure. (**b**) Dispersion relations of the spoof SPPs supported by the structures of varying width *h*. Distributions of the electric field *E*_*y*_ in the *x*-*z* plane for (**c**) even and (**d**) odd modes at different frequencies for the structure with *h* = 6.5 mm.

At microwave frequencies, the corrugated metal strips with near-zero thickness have been demonstrated to support spoof SPPs^36^,^37^,^38^,^39^,^40^,^41^. Here we employed such strips to construct the desired plasmonic structures. Figure 1a shows a schematic illustration of the structure consisting of metal strips corrugated symmetrically by groove arrays and adhered on a thin dielectric layer. The groove width is 0.2 mm and the dielectric layer thickness is 0.8 mm. The period of the groove arrays and the width of the center metal part terminating the grooves are all equal to 0.4 mm. All these geometry parameters are fixed throughout the work. Under the excitation of *x*-polarized waves (the electric field is polarized along the *x* direction), the spoof SPPs supported by the two sides hybridize to form even mode and odd mode due to the symmetry of the corrugated strips^39^,^40^. This can be also learned from the dispersion relations in Fig. 1b as well as the field modes in Fig. 1c,d. In the simulation, the dielectric layer has a permittivity of 4.3 and the metal is assumed as PEC. For a given strip width, obviously, the dispersion curves include two branches corresponding to even and odd modes, respectively^39^, and the asymptotic frequencies of two modes are close to each other. Additionally, the asymptotic frequencies can be easily tailored by changing the strip width, providing a freedom to control *k*_*z*_ of the spoof SPPs for exploring novel applications.

Since the dispersion relations lie below the light line [see Fig. 1b], the spoof SPPs are confined and enhanced in the sub-wavelength regions, as well shown by the electric field *E*_*y*_ distributions in Fig. 1c,d. This effect is especially remarkable when the frequency approaches to the asymptotic frequency where *k*_*z*_ reaches the maximum. In this case, if the structure is lossy, the spoof SPPs will be dissipated when propagating along the groove surfaces.

**Figure 2:**
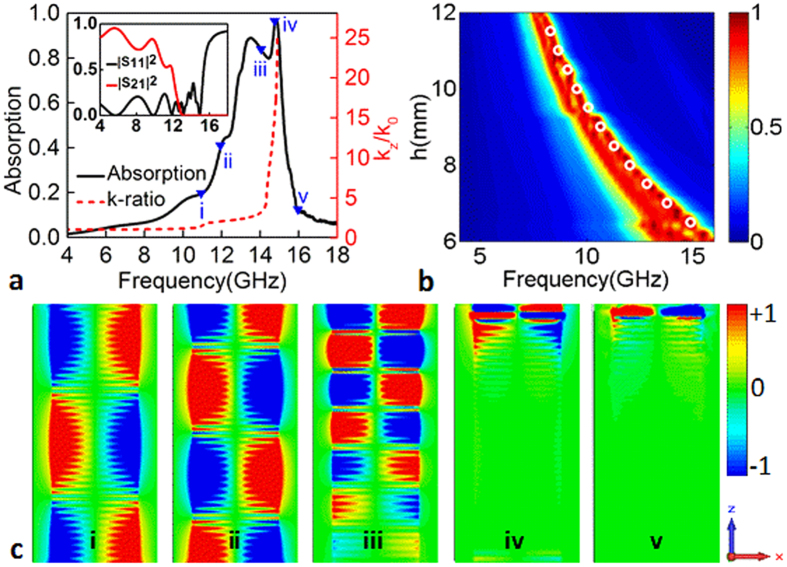
Loss characteristic of the spoof SPPs. (**a**) Absorption along with the ratio *k*_*z*_/*k*_0_ of the spoof SPP as a function of frequency for the structure with strip width *h* = 6.5 mm. The length in the *z* direction is 16.2 mm. The absorption levels at frequencies of (i) 11, (ii) 12, (iii) 13.5, (iv) 14.7 and (v) 16 GHz are highlighted by blue triangles. The inset is the reflection and transmission spectra. (**b**) Absorption spectra for the structures of varied strip width *h*. The locations of the asymptotic frequencies with the different strip widths are indicated by white circles. (**c**) Distributions of the electric field *E*_*y*_ in the *x*-*z* plane at the frequencies of (i) 11, (ii) 12, (iii) 13.5, (iv) 14.7 and (v) 16 GHz.

To verify the above analysis, we take a one-dimensional array (along *x* direction) made of the plasmonic strips as an example. The metal strip width is 6.5 mm and the periods in both *x* and *y* directions are 9.5 mm. The length in the *z* direction is assumed as 16.2 mm (not necessary). The metal has an electric conductivity of 5.8 × 10^7^ S/m and the loss tangent of the dielectric layer is 0.025, introducing the ohmic and dielectric losses, respectively. The incident electric field is polarized along the *x* direction. The absorption is calculated by *A* = 1 − |*S*_11_|^2^ − |*S*_21_|^2^ from the simulated *S* parameters and shown in Fig. 2a. As expected, a distinct absorption behavior with efficiency as high as 90% takes place in the frequency range somewhat below the asymptotic frequency. The similar absorption behavior can be also observed for the cases with other strip widths, as shown by Fig. 2b. It can be furthermore found that the location of the absorption band increases with the strip width *h* decreased from 12 mm to 6 mm, and the upper side of the absorption band is along with the relation of the asymptotic frequency as a function of the strip width highlighted by white circles in Fig. 2b.

To reveal that the absorption is determined by spoof SPPs, we monitored the electric field component *E*_*y*_ at the frequencies of 11, 12, 13.5, 14.7 and 16 GHz, as shown in Fig. 2c. The absorption levels at these frequencies are highlighted by blue triangles in Fig. 2a. Clearly, the spoof SPPs are excited in the corrugated strip array. This can be understood that the uppermost straight metal wires can be approximately considered as a dipole array. In this wise, incident waves can be firstly fed into the corrugated strips by the dipole resonance and then the spoof SPPs are excited via matching the spatial field distribution. However, the spoof SPPs cannot be supported all the same above the asymptotic frequencies, although the dipole resonance on the top wires exists, as shown by the result (v) in Fig. 2c. Since the field distribution of the dipole resonance is anti-symmetric, the odd-mode spoof SPPs are excited in the corrugated strip array. This is different from the plamonic devices fed by waveguides^39^,^41^. Additionally, the structure cannot excite the spoof SPPs with 100% efficiency by partially reflecting the incident wave, and thus realize nearly perfect absorption, as shown in Fig. 2a.

Furthermore, one can better understand the dissipating process of the spoof SPPs by examining the electric field *E*_*y*_ distributions in Fig. 2c. Since *k*_*z*_/*k*_0_ increases with the frequency increased as shown by red dashed line in Fig. 2a, the field confinement is weaker at low frequencies than that at high frequencies. In this case, the propagation length of the spoof SPPs before being completely dissipated is larger at low frequencies than the cases at high frequencies. This is obviously observed from the electric filed *E*_*y*_ distributions at the frequencies of (i) 11, (ii) 12 and (iii) 13.5 GHz. Particularly, since the propagation length at (i) 11 and (ii) 12 GHz is larger than the length (16.2 mm) in the *z* direction, a majority of the spoof SSPs leak out from the other side of the array. As a result, the absorption level is lowered sharply, as shown by the absorption level in Fig. 2a. However, for the case larger than the asymptotic frequency, the spoof SPPs cannot be excited and thus the incident wave is completely reflected, likewise leading a small absorption level.

**Figure 3:**
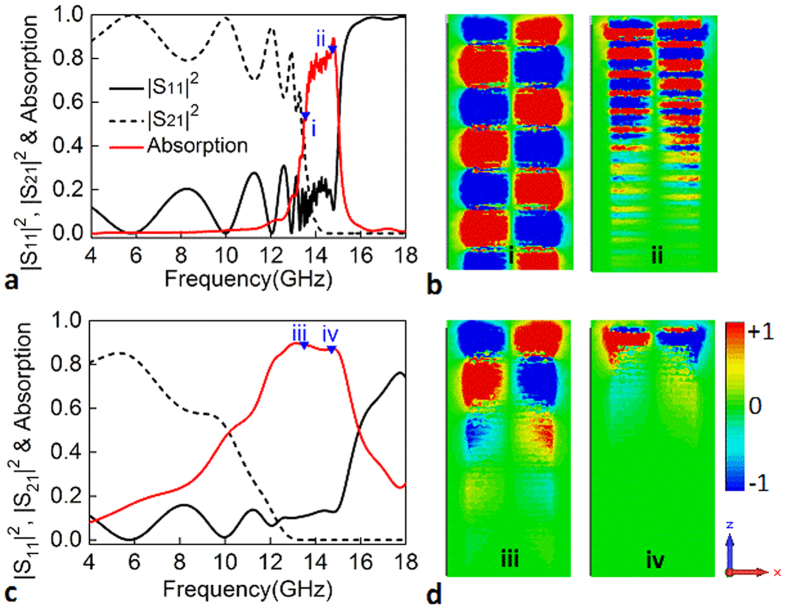
Influence of the loss on the absorption of the spoof SPPs. Absorption along with *S*_11_ and *S*_21_ as a function of frequency for the structures with the loss tangent of 0.001 (**a**) and 0.1 (**c**). The absorption levels at frequencies of (i, iii) 13.5 and (ii, iv) 14.7 GHz are highlighted by blue triangles. (**b,d**) Distributions of the electric field *E*_*y*_ in the *x*-*z* plane at the frequencies of 13.5 and 14.7 GHz.

Additionally, the corrugated strips can be viewed as a set of metallic cut-wires. Such cut-wire resonators may also lead the absorption. To rule out this possibility, we investigate the loss on the absorption properties and the results are presented in Fig. 3. In the simulation, the loss tangent of the dielectric layer is assumed to be 0.001 and 0.1, while other parameters are kept the same used in Fig. 2. By comparing the electric field *E*_*y*_ distributions at the same frequency in Figs 2c,3b,d, one can clearly observe that the spoof SPPs are dissipated gradually as propagating along the strips, and the dissipation distance for the low-loss case is longer than that of the large loss. Particularly, once the propagating distance of the spoof SPPs is smaller than 16.2 mm, the spoof SPPs below the asymptotic frequency can be also strongly dissipated. This can be well demonstrated by the structure with the loss tangent of 0.1, which shows a broadband absorption behavior. It is therefore believed that the absorption of the corrugated strip array results from the propagating spoof SPPs. It can be also concluded that the lossy dielectric layers are necessary to achieve strong absorption for the low-height array.

From above discussions, we can see that the *k*-vector of the spoof SPPs plays a key role determining the absorption behavior, allowing us to control the absorption without considering the constitutive parameters. It will be further shown that the spatial *k*-dispersion engineering of the spoof SPPs allows for realizing the customized absorption, such as broadband absorption and frequency-selective absorption. One notes that, to achieve the strong absorption, the loss tangent of the dielectric layers is assumed to be 0.025 in the following.

### Broadband absorption

Firstly, we show how the broadband absorption is achieved by engineering the spatial *k*-dispersion of the spoof SPPs. It has been shown that the spoof SSPs can be strongly dissipated when *k*_*z*_/*k*_0_ approaches the maximum near the asymptotic frequency, and simultaneously the asymptotic frequency can be tuned by adjusting the groove depth. It is therefore expected that a structure consisting of the grooves with gradient depths can be utilized to achieve broadband absorption, being similar to the trapped rainbow^42^,^43^. Such configuration allows us to gain the maximum *k*_*z*_/*k*_0_ at different frequencies in distinct spatial positions, that is to say, to control the spatial *k*-dispersion of the spoof SPPs. Additionally, the gradient configuration benefits the *k*-vector matching, thus the excitation efficiency of the spoof SPPs as well as absorption can be improved.

**Figure 4:**
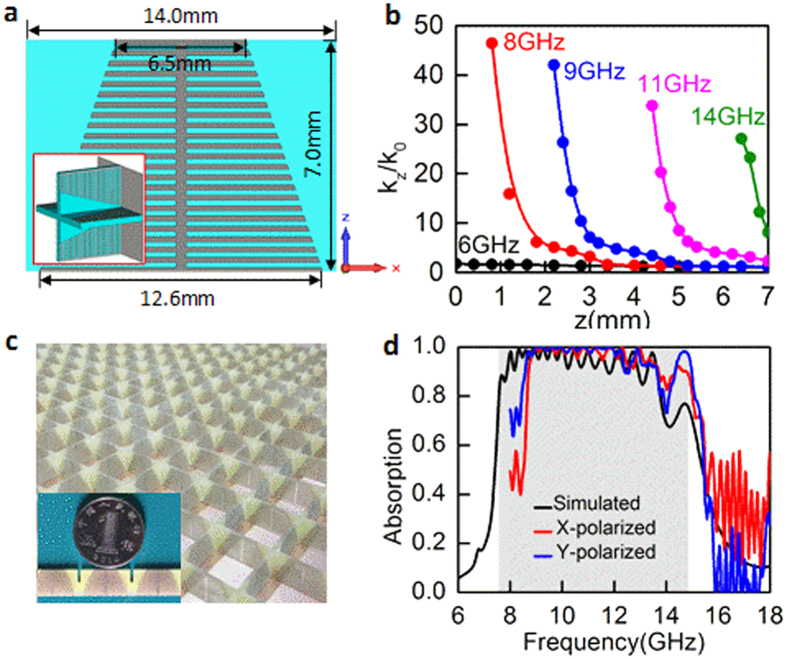
Spatial *k*-dispersion engineering of the spoof SPPs for broadband absorption. (**a**) Design of the unit cell of a broadband absorber. The groove depth decreases linearly from bottom to top. Inset is the three-dimensional schematic of the unit cell. (**b**) Spatial *k*-dispersion relations of the spoof SPPs at different frequencies. (**c**) Photograph of the fabricated sample. (**d**) Comparison between the simulated and measured absorption spectra. The theoretical bandwidth from 7.5 to 14.7 GHz is highlighted by the gray region.

Here we study a structure made of serially connected grooves with linearly varied depth, as shown in Fig. 4a. To gain the polarization-insensitivity response, the plasmonic strips are nested perpendicularly to form a two-dimension array. A metal layer is used on the bottom to prevent the spoof SPPs leak out, as shown by the inset in Fig. 4a. To make the absorber work within 8–18 GHz, the metal strip width is linearly varied from 12.6 to 6.5 mm. The period of unit cell in both *x* and *y* directions is 14 mm and the height in *z* direction is 7 mm. For this gradient configuration, *k*_*z*_ of the spoof SPPs is spatially dispersed along *z* direction. It should be stressed that the linear variation of the groove depth is chosen here just to serve as an example, not necessary.

The spatial *k*-dispersion relations at the frequencies of 6, 8, 9, 11 and 14 GHz are plotted in Fig. 4b. Since the metal strip width decreases from bottom to up, the asymptotic frequency of the spoof SPPs supported by the top is larger than that supported by the bottom and thus *k*_*z*_/*k*_0_ decreases with the height *z* increased from 0 to 7 mm at a given frequency. Specially, *k*_*z*_/*k*_0_ is ~1 at 6 GHz which is smaller than the asymptotic frequency 7.6 GHz related to the maximum metal strip width of 12.6 mm at the position *z* = 0. Therefore, the corresponding absorption level is very small, as shown in Fig. 4d. However, for the frequencies larger than 7.6 GHz, *k*_*z*_/*k*_0_ can reach the maximums at distinct spatial positions. In this case, the spoof SPPs firstly propagate along the groove surfaces from top to bottom and then are remarkably dissipated when reaching the position *z* where *k*_*z*_/*k*_0_ reaches the maximum. Since such condition is fulfilled at numerous frequencies in the range of 7.6–14.7 GHz, the high-efficiency absorption can be theoretically achieved in this frequency band resulting from the overlapping mechanism, as highlighted by the gray region in Fig. 4d. One should note that the maximum value of *k*_*z*_/*k*_0_ is meaningless itself at each frequency.

The absorption as a function of the frequency under normal incidence is plotted in Fig. 4d. Obviously, the absorption behavior consistent with the theoretical result is achieved, providing a validity for the concept of spatial *k*-dispersion engineering for broadband absorption. Nevertheless, we find that the absorption band of more than 90% lies within the gray region highlighting the theoretical bandwidth of 7.6–14.7 GHz. This means that the achieved absorption bandwidth is relatively narrow with respect to the theoretic result. Since the spoof SPPs are supported by several adjacent grooves instead of a single groove [see Fig. 2c], only ones with the asymptotic frequencies somewhat less than 14.7 GHz and larger than 7.6 GHz can be excited, resulting in a slightly narrow bandwidth. In addition, at the frequencies approaching the upper side of the theoretic absorption band, the absorption level is low compared with others. We attribute this to the factor that the spoof SPPs cannot be highly-efficiently excited at these frequencies due to the large *k*-mismatching at the spatial position *z* = 7 mm, as shown by the spatial *k*-dispersion relations in Fig. 4b.

The experimental demonstration of the broadband absorption was performed. The fabricated sample is shown in Fig. 4c. The dimension of the sample is 196 × 196 mm^2^, including of 196 unit cells. The absorption spectra under *x*- and *y*-polarized incidences have been measured from 8 to 18 GHz in a microwave anechoic chamber and are plotted in Fig. 4d. We see that the measured results for both *x*- and *y*-polarized incidences display a broad absorption band, satisfying the result analyzed by the spatial *k*-dispersion engineering. Compared with the simulated result, however, the measured absorption spectra slightly shift towards high frequencies.

**Figure 5:**
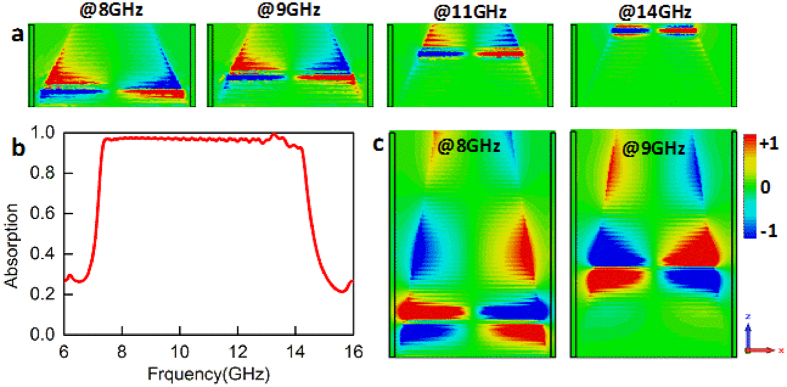
Analysis on the broadband absorption mechanism. (**a**) Electric field *E*_*y*_ distributions in the *x*-*z* plane for the structure in Fig. 4 at the frequencies of 8, 9, 11 and 14 GHz. (**b**) Absorption spectrum of the broadband absorber with the height of 20.6 mm. (**c**) Electric field *E*_*y*_ distributions in the *x*-*z* plane at the frequencies of 8 and 9 GHz.

To further reveal the absorption process, the distributions of the electric field *E*_*y*_ at different frequencies were monitored in the *x*-*z* plane and shown in Fig. 5a. Obviously, at different frequencies the electric field *E*_*y*_ is confined on the distinct spatial positions and the location in the *z* direction shifts gradually from bottom to top with the frequency increased. One can also learn that the field-confined positions are consistent with the locations where *k*_*z*_/*k*_0_ reaches the maximum [see Fig. 4b]. To more clearly display the absorption mechanism of the propagating spoof SPPs, we considered another structure with height in the *z* direction of 20.6 mm. Other parameters are kept as the same values in Fig. 4. The absorption efficiency and the electric field *E*_*y*_ distributions are shown in Fig. 5b,c, respectively. One can see that the spoof SPPs propagate from top to bottom and the wavelength gradually becomes gradually small. This is consistent with that *k*_*z*_ increases from top to bottom, as shown in Fig. 4b.

### Frequency-selective broadband absorption

**Figure 6:**
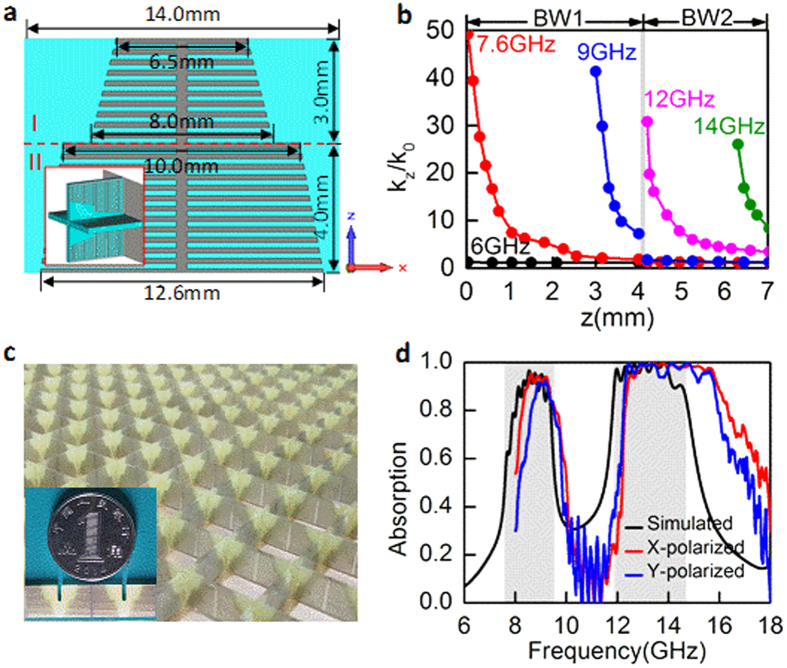
Spatial *k*-dispersion engineering for frequency-selective broadband absorption. (**a**) Design of the unit cell of a frequency-selective broadband absorber. The groove depth decreases linearly from bottom to top with an abrupt change at *z* = 4 mm. The panel of unit cell in the *x*-*z* plane is divided into the top region I and the bottom region II by the interface line. Inset is the three-dimensional schematic of the unit cell. (**b**) Spatial *k*-dispersion relations at different frequencies. Two theoretical bandwidths termed as BW-1 and BW-2 are marked in the top axis. (**c**) Photograph of the fabricated absorber. (**d**) Comparison between the absorption spectra respectively simulated and measured from the fabricated sample. Two theoretical bandwidths of 7.6–9.5 GHz and 11.9–14.7 GHz are highlighted by gray regions.

In order to achieve frequency-selective broadband absorption, we studied a stepped structure with the strip width varied from 10 to 8 mm at *z* = 4 mm and divided into two regions I and II, as shown in Fig. 6a. Such a stepped configuration cannot support the spoof SPPs with the asymptotic frequencies within the band of 9.5–11.9 GHz, which is associated with the strip width varied from 10 to 8 mm. The spatial *k*-dispersion relations for the stepped structure is plotted in Fig. 6b. In the frequency band covering from 9.5 to 11.9 GHz, the spoof SPPs can propagate in the top region I but are reflected by the upper edge (i.e. *z* = 4 mm) of the region II. However, the absorption condition can be fulfilled all the same in other bands of 7.6–9.5 GHz and 11.9–14.7 GHz, as marked by BW-1 and BW-2 in top axis of Fig. 6b. As a result, such spatial *k*-dispersion characteristic allows us to achieve frequency-selective broadband absorption.

Because of the backed metal plate, the absorption is calculated by *A* = 1 − |*S*_11_|^2^ from the simulated *S*_11_ and plotted in Fig. 6d. It can be seen that the absorption band is broken into two sub-bands by a high reflection window, achieving a frequency-selective absorption behavior. This is in a good agreement with the result obtained by the spatial *k*-dispersion engineering, which is highlighted by the gray regions in Fig. 6d. Additionally, we noted that the absorption level in the band of 7.6–9.5 GHz is somewhat low with respect to that in the other band from 11.9 to 14.7 GHz. We attribute this to the additional *k*-mismatching at the position of *z* = 4 mm, as shown by the gray interface region in Fig. 6b.

The experiment is also performed to achieve the frequency-selective absorption. The fabricated sample is shown in Fig. 6c and the measured absorption under both *x*- and *y*-polarized incidences are plotted in Fig. 6d. As predicated, the measured results display a distinct frequency-selective absorption behavior. One should note that, however, the measured lower absorption bands for the *x*- and *y*-polarized waves display some differences, which may result from the measurement errors. Additionally, the measured results for two polarizations shift slightly towards high frequencies compared to the simulation, as also observed in Fig. 4d. We attribute this to the possible that the true permittivity of FR-4 boards may be smaller than the assumed in the simulation, because the small permittivity can result in a large asymptotic frequency for the spoof SPPs.

**Figure 7:**
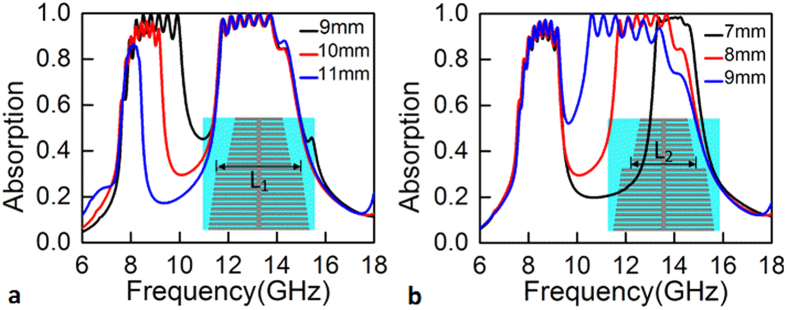
Absorption spectra of the stepped structures. Absorption as a function of frequency with (**a**) *L*_1_ = 9, 10, 11 mm and (**b**) *L*_2_ = 7, 8, 9 mm. The absorption spectrum in Fig. 6 is plotted in (**a**) and (**b**) with *L*_1_ = 10 mm and *L*_2_ = 8 mm, respectively.

The flexibility of the spatial *k*-dispersion engineering in the design of frequency-selective broadband response has also been examined. The results are shown in Fig. 7. In the simulation, the upper strip width *L*_1_ of the region II and the bottom strip width *L*_2_ of the region I are varied, respectively, while other parameters are kept as those used in Fig. 6. It can be observed that *L*_1_ determines the upper side of the absorption band at lower frequencies, while *L*_2_ influents the bottom side of the absorption band at higher frequencies. This is well consistent with the results analyzed by engineering the spatial dispersion of *k*-vector. It is therefore believed that one can easily gain the desired frequency-selective broadband response by the proposed concept.

## Conclusions

In summary, we have demonstrated that the spatial *k*-dispersion relation of spoof SPPs can be engineered to produce customized absorption over a broad frequency band. The field confinement effect within sub-wavelength regions of the spoof SPPs is extremely remarkable near the asymptotic frequency, which allows the corresponding exciting device to operate as an absorber by introducing suitable loss. Combining this ability with the controllable dispersion relations of the spoof SPPs, we took two linearly gradient configurations as examples to show how to gain broadband and frequency-selective broadband absorption by engineering the spatial *k*-dispersion relations of the spoof SPPs, respectively. Unlike the nearly perfect absorbers^4^,^17^,^19^,^22^,^29^,^44^, the absorption efficiency may not be as high as 100% because of the *k*-mismatching between the spoof SPPs and the space wave. However, the advantage of this concept is that it does not require the complex process of retrieving the constitutive parameters and subsequent optimization. Additionally, the spatial *k*-dispersion engineering provides a different perspective on the previous multiband/broadband metamaterial absorbers^18^,^20^,^21^,^23^,^24^,^44^,^45^,^46^,^47^. We further expect several applications of the general principle of the spatial *k*-dispersion engineering in manipulating propagating waves, such as anomalous refraction/reflection^48^,^49^,^50^ and polarization manipulation^51^.

## Methods

### Simulations

Electromagnetic simulations are performed using a commercially available software package, CST Microwave Studio. The dispersion relations are calculated using the Eigen-mode solver with periodic boundary conditions along the *x, y* and *z* directions. The *S* parameters are simulated using the Transient solver and the electric field distributions are monitored simultaneously. In the simulation, periodic boundary conditions in the *x* and *y* directions are used, and open boundary conditions in the *z* direction.

### Fabrication

The samples are fabricated using the standard PCB photolithography. The commercial FR-4 boards are used as the dielectric layers and the 17-μm-thick copper films as the metal parts. The strips with copper grooves were firstly fabricated and then assembled together to obtain samples. All the samples include 14 × 14 unit cells with a dimension of 198 mm × 198 mm.

### Measurements

The experimental study of absorption is performed by the arch measurement system in a microwave anechoic chamber. The system is based on an Agilent 8720ET network analyzer with a pair of broadband horn antennas working in the frequency range of 8–18 GHz. Two antennas are used for transmitting and receiving signals, respectively. In the measurement, the incident angle is 4 degrees and the reflection from a metal plate with the same size as the samples is firstly used for normalization. The absorption is then calculated by *A* = 1 − |*S*_11_|^2^, where *S*_11_ is the measured reflection coefficient from the sample backed by a metal plate. By rotating the orientation of the samples by 90 degrees with respect to the antennas, the absorption is measured for *x*- and *y*-polarized incidences.

## Additional Information

**How to cite this article**: Pang, Y. *et al*. Spatial *k*-dispersion engineering of spoof surface plasmon polaritons for customized absorption. *Sci. Rep.* **6**, 29429; doi: 10.1038/srep29429 (2016).
